# The profiling of phenolic compounds in archive Tokaj wines using liquid chromatography and antioxidant activity analysis

**DOI:** 10.1016/j.fochx.2025.102897

**Published:** 2025-08-07

**Authors:** Pavlína Moravcová, Ivan Špánik, Jan Škop, Aleš Horna, František Švec, Adriano de Araújo Gomes, Dalibor Šatínský

**Affiliations:** a Department of Analytical Chemistry, Faculty of Pharmacy in Hradec Králové, Charles University, Ak. Heyrovského 1203, Hradec Králové 500 05, Czech Republic; b The Institute of Analytical Chemistry, Faculty of Chemical and Food Technology, The Slovak University of Technology in Bratislava, Radlinského 9, Bratislava 812 37, Slovakia; c Institute of Nutrition and Diagnostics Pardubice, Sakařova 1400, 530 03 Pardubice, Czech Republic; d Universidade Federal do Rio Grande do Sul, Instituto de Química, 90650-001 Porto Alegre, RS, Brazil

**Keywords:** Tokaj wine, Phenolic profiling, Antioxidant capacity, Chromatography, Principal component analysis, SIMCA, Authenticity

## Abstract

Archive Tokaj wines from the vineyards in the Slovak part of the Tokaj region have been characterized in terms of the content of phenolic compounds specific to that type of botrytized wine. More than 60 archive samples (1959–2017) were evaluated in terms of phenolic profile. Eighteen individual phenolic compounds were quantified by UHPLC-DAD method. The total concentration of phenolic compounds in the wines ranged from 58.28 to 302.57 mg/L. The most abundant compound in the phenolic profile was caftaric acid with average values of around 60 mg/L followed by catechin ranging from 2.24 to 49.35 mg/L, gallic acid with a mean concentration of 14.65 mg/L, and vanillic acid. Statistical evaluation using PCA and SIMCA model showed significant correlations between the phenolic profile, botrytized/non-botrytized wines, and their origin. Total antioxidant capacity determination using a CoulArray detector proved the correlation between the “putňa” number of Tokaj selections and antioxidant activity of wines.

## Highlights

• HPLC method for quantification of 18 phenolic compounds in 62 Tokaj wines was developed.
• PCA statistical evaluation was performed on the data set containing 11 polyphenols.
• SIMCA model identified key contributors to botrytized and non-botrytized wines discrimination.
• Catechin, Gallic, 4-hydroxybenzoic, and Caftaric acid authenticated the Tokaj wines.
• Correlation between phenolic profile and amount of noble rotten berries was provided.

## Graphical abstract

**Figure:**
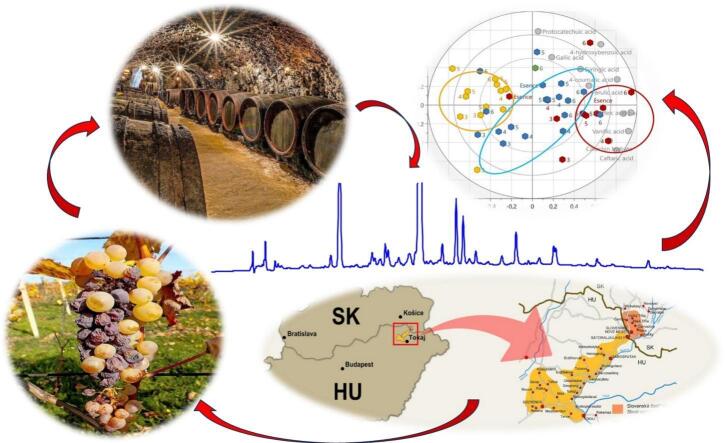

## 1 Introduction

Tokaj wines represent a group of botrytized wines produced from overripe grapes infected with the noble rot *Botrytis cinerea*. This category of wines is characterized by a higher residual sugar content and unique chemical composition (Magyar, 2011). The characterization of Tokaj selection wines is expressed in the number of “putňa” in relation to the number of baskets with a volume of 28 L meaning 20–25 kg of botrytized berries added to a 136 L barrel of the wine. Typically, Tokaj wines were produced as 3-, 4-, 5-, and 6 putňa sugar levels in the strictly defined wine region, which falls under two European countries, Hungary (5800 ha) and Slovakia (929 ha) (Khvalbota et al., 2021a). In 2013, the traditional 3–6 putňa system was abolished and replaced by four categories based on residual sugar content of 60, 90, 120, and 150 g/L corresponding to 3, 4, 5, and 6 putňa. The specific geographical, and climatic conditions of the Tokaj wine region include red clay soil and volcanic tuff subsoil, foggy mornings, and warm, dry, and sunny days, which enable the production of grapes with distinctive terroir (Makra et al., 2009). Predicting and distinguishing the geographical origin of food products is an approach to protect the reputation and value of the specific products, as evidenced especially in the European Union under the classifications “Protected Designation of Origin” and “Protected Geographical Indication” (McGhie & Rowan, 2012). Therefore, winegrowing and vinification procedures in Tokaj vineyards are regulated by laws of the European Commission (Commission Regulation No. 607/2009, 2009) and by the national legislation of both involved countries, which falls under the Tokaj wine region (Furdíková et al., 2019). Differentiation of the geographical origin of food products and their chemical composition is an essential attribute that can contribute to preventing food adulteration, and help to protect consumers from the risks associated with adulteration and counterfeit wine (Regulation (EU) 2024/1143). The most typical topics of counterfeiting related to wine are the indication of grape variety, geographical origin, and vintage year (Merkytė et al., 2020; Temerdashev et al., 2024; Regulation EU, 2024; Gajek et al., 2021; Gao et al., 2022).

Wine quality and authenticity assessment are usually based on sensory and chemical analysis. On that score, variations of chemical substances originating from grape berries or formed during winemaking can be a helpful tool for wine differentiation. According to previous studies, wines can be evaluated and differentiated based on the profile of polyphenol substances, biogenic amines, or volatile organic compounds (Costantini et al., 2019; Furdíková et al., 2020; Galgano et al., 2011; Luo et al., 2024; Moreira et al., 2024; Ordóñez et al., 2017). The phenolic compounds can provide relevant information for assessing wine authenticity and can be successfully used as chemical markers, as the geographical origin, grape variety, winemaking process, and viticulture practices significantly influence the quantity and quality of these substances (Bambina et al., 2024; Lamplíř & Pavloušek, 2013; MavricScholze et al., 2025; Merkytė et al., 2020; Uzkuç et al., 2025). Moreover, the phenolic composition of botrytized wines is affected by noble rot *Botrytis cinerea*, by the enzymatic activity of *Botrytis* and the metabolic response of the plant to noble rot infection that plays the main part in the formation of the unique phenolic profile of Tokaj wines. The process by which grapes achieve these desirable attributes is due to the oxidation of aromatic metabolites through enzymatic reactions from laccase-catalyzed oxidation, producing new compounds e.g. furfural, phenylacetaldehyde, and lactones, among others (Bailly et al., 2006; Magyar, 2011). Different laccases are induced by grape juice, gallic acid, and p-coumaric acid. In addition, pectin may augment laccase synthesis in the presence of phenolic compounds. In wine, laccases oxidize a wide range of important grape phenolics, for example: *p*-, *o*-, and some *m*-diphenols, diquinones, anthocyanins, tannins, and a few non-phenolics such as ascorbic acid (Jackson, 2008). This may partially explain the comparatively high phenolic content of botrytized wines and the decrease of hydroxycinnamic acids. Oxidation products of the reaction of glucose oxidase with glucose further react with catechins and proanthocyanidins and affect the visual quality of wine, while polyphenol oxidase directly affects a wide range of polyphenols (Blanco-Ulate et al., 2015; Magyar & Soós, 2016). The group of wine phenolics comprises compounds with various chemical structures and functions and is divided into two main classes, non-flavonoids and flavonoids (Jackson, 2008). Non-flavonoids include hydroxybenzoic acids, hydroxycinnamic acids, and stilbenes, and mainly originate from the grape skins (Sun et al., 2006). In terms of quantity, the hydroxycinnamic acid derivatives are the most abundant non-flavonoid compounds in white wines. The caftaric, coumaric, caffeic, and ferulic acid are the major hydroxycinnamic derivatives in white wines that can serve as substrates for polyphenol oxidase and play a key role in oxidative browning. In small amounts, oxidized derivatives can contribute to the slightly yellow coloring of Tokaj wine. Among grape non-flavonoid compounds, hydroxybenzoic acids, gallic, protocatechuic, syringic, and vanillic acid, occur in lower concentrations than cinnamic derivates. Stilbenes, resveratrol, and piceid are usually a minor group of the non-flavonoids of white wines, but during a fungal invasion, plants react by increased production of stilbenes. However, in the last stage of noble rot infection, stilbenes act as phytoalexins resulting in a relatively low final concentration of resveratrol and piceid in botrytized wines (Jackson, 2008; Moreno-Arribas & Polo, 2009). During infection, laccases are one of the most significant enzymes produced by *B. cinerea* and are suspected to inactivate antifungal phenols, such as pterostilbene and resveratrol in grapes (Jackson, 2008; Pezet et al., 1991). Moreover, several pathogenic fungi can either inhibit or destroy phytoalexins such as resveratrol. The major flavonoid compounds found in wine are either flavonols, flavan-3-ols, or anthocyanins. In terms of quantity, flavonoids typically dominate among phenols in red more wines than in white wines. Most flavonoids in white wines are represented by catechins (epicatechin, catechin, and epicatechin gallate) (Jackson, 2008, Moreno-Arribas & Polo, 2009).

In this work, we have characterized Tokaj wines in terms of their unique phenolic composition, antioxidant capacity, and provided the contribution to the authenticity assessment of botrytized wines. The two general approaches are usually applied to examine and quantify the content of wine phenolics, the estimation of total phenolic content and antioxidant capacity, or the separation of individual phenols and their subsequent detection. The reverse-phase liquid chromatography with C-18 or equivalent reversed stationary phases with UV/DAD, FLD, and MS detectors dominates among methods used for the analysis of individual phenolic compounds in wine. Despite the structural elucidation, even at very low concentrations provided by the MS, UV detection remains the method of choice due to the natural absorbance of phenolics in the UV region (Stalikas, 2007). In opposition to the specific analysis of individual phenolics is the determination of global index e.g., total polyphenols, and is mainly achieved by spectrophotometric methods like the Folin-Ciocalteau assay Compendium of International Methods of Wine and Must Analysis, 2021, an official standard method for some food products, such as wine (Naczk & Shahidi, 2006). These methods suffer from several drawbacks like matrix effects and interfering compounds resulting in an overestimation of antioxidant capacity. To overcome these issues the electrochemical methods are applied to study antioxidants, specifically the coulometric array detector coupled to HPLC or FIA systems (Escarpa & González, 2001).

Only a few reports on the characterization of volatile organic compounds in Tokaj wines were presented (Furdíková et al., 2020; Furdíková et al., 2021; Khvalbota et al., 2021b; Machyňáková et al., 2019; Sádecká & Jakubíková, 2020; Vyviurska & Špánik, 2020). Nevertheless, none of them dealt with the profiling of phenolic compounds. Therefore, the main aim of this study was to develop and validate a new UHPLC method for the determination of eighteen phenolic compounds in Tokaj wines. These eighteen substances are one of the most abundant phenolic compounds in white wines and have been successfully employed as markers for the identification of the origin of wine or its cultivar (Jackson, 2008; Kupsa et al., 2017; Lamplíř & Pavloušek, 2013; Pour Nikfardjam et al., 2007). The chromatography conditions, such as the type of stationary phase based on advanced fused core column technology and choice of the mobile phase covering different gradient elution profiles were optimized to achieve fast and efficient separation with high resolution of phenolic compounds. The deeper insight into the relationship and correlation among the profile of phenolic compounds and vintage, the amount of noble rotten berries (putňa), and various producers and vinification techniques using statistical analysis based on PCA and SIMCA model was a secondary objective of this work. Furthermore, the total antioxidant capacity and the correlation among the phenolic compounds were estimated. Our study is the first that contributes to the characterization of Tokaj wines based on the profile of phenolic substances and identify the compounds specific to that type of botrytized wine.

## 2 Materials and methods

### 2.1 Tokaj wines

Sixty-two samples of archived Tokaj wines (1959–2017) originating from the Slovak part of the Tokaj wine region were analyzed. These samples were obtained from three different wineries, namely Tokaj and Company (located in Malá Tŕňa), Ostrožovič (located in Veľká Tŕňa), and Tokaj Zlatý strapec (located in Viničky). Tokaj wines were vinified from a few white grape cultivars (namely Yellow Muscat, Furmint, and Lipovina). Tokaj specialty wines included Tokaj Selections, Tokaj Esence, Fordítás, Máslás, and Samorodni dry and sweet wines. Tokaj Selections covered the putňa index in the range from three to six. The wines from each category contained a minimum sugar and extract content of 60, 90, 120, and 150 g/L sugar, respectively. Tokaj Essence was produced only from noble-rotted berries with a very high sugar content of around 450 g/L. Tokaj Samorodni is classified as sweet or dry depending on the number of noble-rotted berries and the sugar content of the must (Magyar, 2011). The Czech wines from the South Moravia region: Riesling (Archlebov winery), Müller Thurgau and Chardonnay (Sádek winery), (Kupsa et al., 2017; Lamplíř & Pavloušek, 2013), were used for comparison of phenolic profiles. The list of archival samples is presented in the Supplementary Material, Table 1S. Samples were kept in the refrigerator at approximately 4 °C until further analysis.

**Table 1:** The analytical characteristics – system suitability test of the validated UHPLC-DAD method used for the identification and quantification of phenolic compounds. Peak symmetry and peak resolution were calculated by LabSolutions software.

Phenolic compound [no. in chromatogram]	Detection wavelength [nm]	Retention time [min]	Retention time RSD [%] 1	Peak resolution	Peak symmetry	Peak area repeatability RSD [%] 2
Gallic acid [1]	280	1.47	0.09	–	1.31	1.05; 0.64; 0.57
Protocatechic acid [2]	280	2.55	0.05	14.41	1.14	1.77; 0.68; 0.49
4-hydroxybenzoic ac. [3]	280	3.97	0.09	16.11	1.06	1.44; 0.68; 0.43
Caftaric acid [4]	320	4.18	0.11	2.17	1.07	2.86; 0.99; 0.66
Catechin [5]	280	5.58	0.14	13.84	1.03	3.91;1.65; 1.22
Vanillic acid [6]	280	5.85	0.13	2.55	0.99	0.70; 0.29; 0.27
Caffeic acid [7]	320	6.08	0.13	1.94	1.04	0.92; 0.21; 0.50
Chlorogenic acid [8]	320	6.81	0.13	6.68	0.97	2.78; 0.31; 0.35
Syringic acid [9]	280	7.54	0.12	6.69	0.97	0.81; 0.21; 0.39
Epicatechin [10]	280	8.38	0.11	7.17	0.95	2.34; 1.01; 1.10
4-coumaric acid [11]	320	8.68	0.10	2.32	1.01	0.54; 0.24; 0.59
Fertaric acid [12]	280	10.47	0.08	12.39	0.99	0.77; 0.26; 0.34
Ferulic acid [13]	320	11.05	0.08	3.99	0.97	1.58; 0.42; 0.32
Sinapic acid [14]	320	12.45	0.07	9.82	0.96	2.07; 0.60; 0.49
Piceid [15]	320	12.99	0.06	3.71	0.96	0.98; 0.50; 0.38
Rutin [16]	280	14.97	0.07	16.20	0.98	1.82; 0.64; 0.70
2-hydroxy-4-methoxybenzoic acid [17]	280	15.25	0.07	1.85	0.99	3.57; 1.03; 1.03
Resveratrol [18]	320	17.46	0.06	10.98	1.37	2.57; 1.27; 0.99

1 Number of replicates, *n* = 6.

2 Calculated from six injections of standard mixture at concentrations of 1, 5, and 10 mg/L.

### 2.2 Materials

Gallic acid, protocatechuic acid, 4-hydroxybenzoic acid, catechin hydrate, vanillic acid, caffeic acid, chlorogenic acid, syringic acid, epicatechin, 4-coumaric acid, fertaric acid, ferulic acid, sinapic acid, piceid, rutin, 2-hydroxy-4-methoxybenzoic acid, and resveratrol were purchased from Sigma-Aldrich (St. Louis, USA) with purity ranging from 90 % to 99 %; caftaric acid with purity of 95 % was purchased from Dalton Pharma Services (Toronto, Canada). HPLC gradient grade solvents acetonitrile (ACN) and methanol (MeOH) were purchased from Fischer Chemical (United Kingdom). Analytical grade phosphoric acid 85 % was purchased from Merck (Germany). Water was purified to ultrapure level in the Milli-Q system (Millipore, USA).

The stock standard solutions of all phenolic compounds were prepared by dissolving 1 mg of each standard individually in 1 mL MeOH. All stock solutions were stored in the refrigerator at 4 °C. Working standard solutions were prepared by diluting the standard stock solutions with MeOH. For the method validation, calibration standard solutions were prepared freshly on the day of analysis by diluting the stock standard solutions with MeOH to obtain eight concentration levels (0.250, 0.5, 1, 2, 5, 10, 20, and 40 mg/L). The accuracy of the method was verified as recovery and was performed by preparing eight solutions: six solutions of spiked Tokaj wine (final concentration of phenols 5 mg/L), two solutions of a none spiked of Tokaj wine (no.15) with added 100 μL MeOH, and two standard solutions of phenolic compounds (5 mg/L).

### 2.3 Instrumentation

The chromatographic analysis of phenolic compounds was carried out using the UHPLC Nexera X2 system (Shimadzu Corporation, Japan), which consisted of a DGU-20 A5R degassing unit, a CBM-20 A communication module, an LC-30 AD solvent delivery system, a CTO-20 AC column oven, and an SPD-M30A diode array detector. The LC Lab-Solution chromatography software (Shimadzu Corporation, Japan) was used for data processing.

### 2.4 Chromatographic conditions

Phenolic compounds were separated on Kinetex Polar C18 100A core-shell analytical column (150 × 3.0 mm), 2.6 μm particle size (Phenomenex, Torrance, CA, USA) with a total run time of 22.5 min. Samples of Tokaj wine in a volume of 1 μL were injected directly into the column and eluted at a flow rate of 1.0 mL/min and a column temperature of 50 °C in a gradient mode comprising 0.1 % aqueous phosphoric acid (solvent A) and acetonitrile (solvent B). The linear gradient program was 2 to 19 % *v*/v B in A in 20.0 min, 19 to 2 % v/v B in A in 0.2 min, followed by column equilibration in this mobile phase for 2.3 min. The detection wavelengths were set at 280 and 320 nm according to the spectral characteristics of the analyzed compounds.

### 2.5 The total antioxidant activity

The screening of the total antioxidant activity of Tokaj wines was performed in a flow injection analysis (FIA) mode without a chromatographic column in the LC system. The flow system consisted of two solvent delivery pumps (Model 583, ESA Inc., Chelmsford, MA, USA) with an autosampler (Model 543 HPLC, ESA Inc., Chelmsford, MA, USA) coupled to a CoulArray electrochemical detector (Model 560 A, ESA Inc., Chelmsford, MA, USA). The CoulArray detector included one flow cell (Model 6210, ESA Inc., Chelmsford, MA, USA) equipped with four porous glassy carbon working electrodes each with an auxiliary and two dry hydrogen–palladium reference electrodes, operating at potentials of +200 mV, +400 mV, +600 mV, and + 800 mV. Data acquisition and data evaluation were performed using the CoulArray® Data Station. For the determination of the total antioxidant activity, the following measurement settings were used: the carrier solution consisting of 0.05 mol/l KH_2_PO_4_ in water and 10 % ACN (pH = 4.8) was delivered at a flow rate 1.0 ml/min, the temperature was maintained at 30 °C, and the total run time of the analysis was the 70 s. All wines were diluted 250 times in carrier solution before analysis, and the injection volume was 10 μL. The total antioxidant capacity was monitored and expressed as the mean of the total electric charge in microcoulombs (μC), by integrating the peak area of the response of the four working electrodes in series.

## 3 Results and discussion

### 3.1 Optimization of chromatographic analysis

The major challenge we faced at the beginning of the method development was related to the chemical structural similarity of some major phenolic compounds and consequently, their similar retention, while other flavonoid compounds significantly differed in longer retention times. We encountered many challenges in the separation and quantification of these compounds, namely epicatechin and vanillic acid, fertaric acid and rutin, chlorogenic acid, and catechin. To alleviate the problem of insufficient resolution and to avoid the overlapping of critical pairs, different operating conditions (gradient elution mode and different gradient profiles, temperature, and flow rate) and several columns with different stationary phase chemistries were studied. Specifically, C18 with core-shell (Kinetex EVO, XB, and Polar) and fully porous particles (Luna Omega Polar and PS), also C18 with modifications with excellent selectivity for isomers and structural analogs (YMC Triart ExRS), RP-Amide (core-shell Ascentis Express), and pentafluorophenyl with core-shell (Kinetex) and fully porous particles (YMC Triart). All columns had a length of 150 mm, 3.0 or 4.6 mm i.d., and particle size less than 3 μm. Based on the previous comparative study the following three columns were further investigated because of their potentially desired selectivity and the beneficial effects of core-shell particles, such as increasing column efficiency while decreasing run time: PFP Triart (150 × 4.6 mm; 3 μm), Kinetex 1.7 μm F5 (150 × 2.1 mm; 1.7 μm), and Kinetex 2.6 μm Polar C18 (150 × 3.0 mm; 2.6 μm). Two of the three columns tested contained a fluorinated (PFP) stationary phase with dual-mode retention behavior owing to the presence of both polar and non-polar centers. Their selectivity is exceptional due to the different forces resulting from hydrophobic, π-π, and dipole-dipole interactions and the PFP phase has remarkable results in the separation of aromatic compounds, such as polyphenols. However, in our case, we did not observe the improved separation efficiency for the complex phenolic profile of Tokaj wine. The other tested stationary phase providing good results was the Kinetex 2.6 μm Polar C18 column, which combines the alkyl C18 ligand and embedded polar functional groups. In contrast to the traditional C18, the absence of any added polar group, we observed a significant improvement of the peak shape of polar compounds and enhanced polar selectivity allowing the efficient separation of polar and non-polar compounds simultaneously. The stationary phases were examined under an optimized gradient program for each column, and the flow rates were tested in the range of 0.4 mL/min to 1.0 mL/min considering the diameter, length, and particle size of each column. Acetonitrile and aqueous phosphoric acid solution were used as the mobile phase components during optimization. The use of 0.1 % phosphoric acid instead of water as the aqueous mobile phase components resulted in the reduction of the ionization of the phenolic hydroxyl group. The two different organic solvents, acetonitrile and methanol, were compared and methanol had no positive effect on the retention factors. Acetonitrile was chosen for its ability to elute faster, while improving peak symmetry. The temperature of the column oven was tested from 25 °C to 60 °C, the optimum temperature of 50 °C was used for further analysis considering the temperature limit of the column and the undesirable acceleration of elution of the first peak of gallic acid that coeluted with column void volume. The detection wavelength was set at 280 nm to identify hydroxybenzoic acids and flavan-3-ols; hydroxycinnamic acids, and stilbenes, respectively at 320 nm to identify flavonols.

**Fig. 1:**
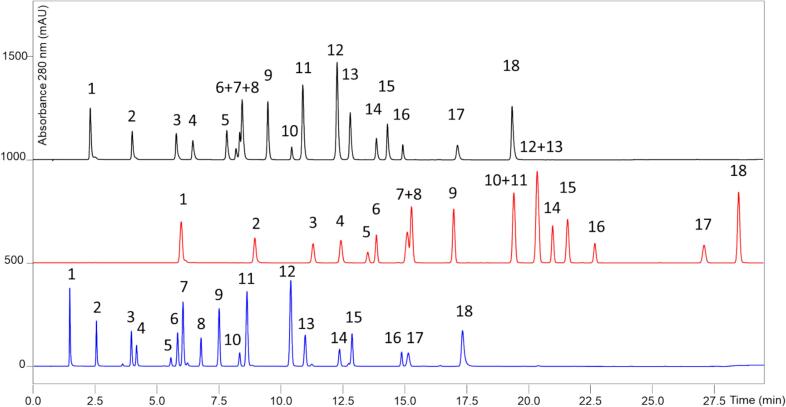
Comparison of the separation of phenolic compounds in standard solution performed on three different stationary phases: black line - Kinetex F5, red line - PFP Triart, and Blue line - Kinetex Polar C18 using the optimal separation conditions for all columns. Detection wavelength 280 nm for all compounds. Peak identification: **1** Gallic acid; **2** Protocatechuic acid; **3** 4-hydroxybenzoic acid; **4** Caftaric acid; **5** Catechin; **6** Vanillic acid; **7** Caffeic acid; **8** Chlorogenic acid; **9** Syringic acid; **10** Epicatechin; **11** 4-coumaric; **12** Fertaric acid; **13** Ferulic acid; **14** Sinapic acid; **15** Piceid; **16** Rutin; **17** 2-hydroxy-4-methoxybenzoic acid; and **18** Resveratrol. (For interpretation of the references to colour in this figure legend, the reader is referred to the web version of this article.)

**Fig. 2:**
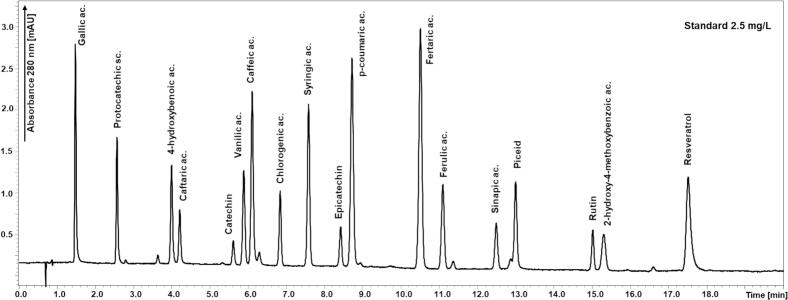
Chromatogram of standard solution at concentration 2.5 mg/L under the validated chromatographic conditions (details in Section 2.4) performed on a Kinetex Polar C18 core-shell column at 50 °C. Acetonitrile and 0.1 % aqueous phosphoric acid used as mobile phase components following to the elution gradient program. Flow rate 1.0 mL/min and a column temperature of 50 °C. Detection wavelength 280 nm.

**Fig. 3:**
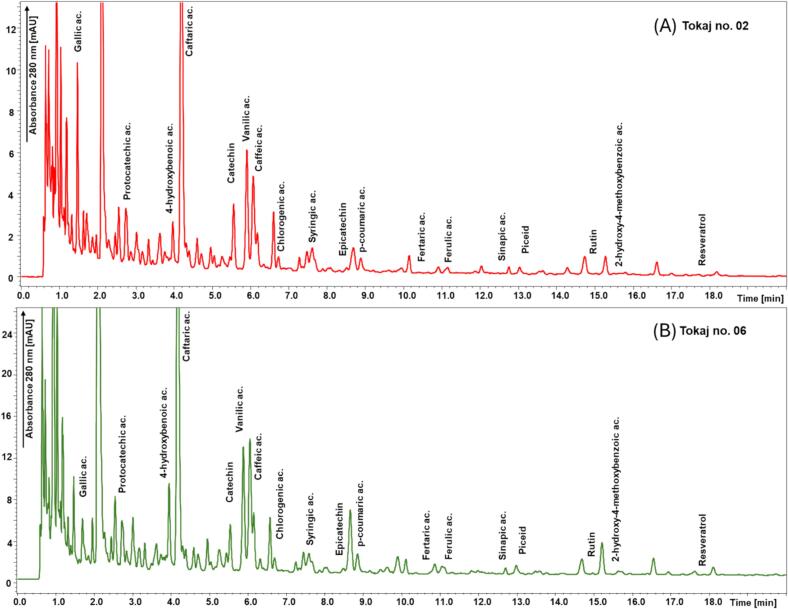
Chromatogram of Tokaj wine sample No. 02 (A) and sample No. 06 (B) under the validated chromatographic conditions. The details of the validated UHPLC-DAD method and conditions of gradient elution are demonstrated in Section 2.4.

In agreement with the results of the chromatographic optimization, the Kinetex Polar C18 column was selected for further validation under the optimized conditions. As shown by the separation of the standard solution in Fig. 1 and the separation of the standard solution under the validated conditions in Fig. 2. These conditions were the most suitable for the analysis of all eighteen phenolic compounds in the shortest time. The chromatograms of two archival Tokaj wines are demonstrated in Fig. 3A and B.

### 3.2 Validation

The performance of the developed UHPLC-DAD method was evaluated in terms of linearity, precision, the limit of detection, the limit of quantification, and accuracy with respect to ICH guidelines. The method validation procedure included a chromatography system suitability test (SST). The following parameters were evaluated using the SST of the chromatographic method: peak resolution (Rs), peak symmetry (S), retention time repeatability (t_R_), and peak area repeatability (A) (expressed as the relative standard deviation RSD in %). All parameters of the SST were performed and calculated from six replicate injections of the mixed standard solution at three different concentration levels of 1, 5, and 10 mg/L. Details of the SST are summarized in Table 1.

To test the linearity of the detector response, standard calibration solutions were prepared at eight concentration levels, ranging from 0.250 to 40 mg/L for all compounds. The concentration range was proposed according to the concentration of phenolic compounds commonly found in white wine samples. The detection and quantification limits of all phenolic compounds were determined as the lowest concentration of the linearity range while giving a signal-to-noise ratio greater than 3 (S/N = 3), and 10 (S/*N* = 10), respectively, with accuracy within ±10 %. The precision of the proposed method was investigated by the determination of six independently prepared solutions of sample no.15 spiked with a solution of standards, which were not present in this wine, each sample was injected in triplicate. The relative standard deviations of all phenolic compounds ranged from 0.47 to 9.23 %. The accuracy of the method was verified as the recovery after the standard addition procedure for six spiked samples of Tokaj wine no. 15. The data summarized in Table 2 confirm that the percentage recovery values were within the acceptable range from 85.96 ± 1.66 to 118.67 % ± 3.15 %.

**Table 2:** The analytical characteristic of the validated UHPLC-DAD method used for the identification and quantification of phenolic compounds.

Phenolic compound [no. in chromatogram]	Detection wavelength [nm]	Slope 1	Intercept 1	Determination coefficient 1	LOQ (mg/L)	Recovery [%] ± RSD [%]^2^
Gallic acid [1]	280	3186.93 ± 23.94	566.71 ± 368.35	0.99980	0.125	102.93 ± 0.92
Protocatechic acid [2]	280	2092.52 ± 14.45	364.23 ± 222.39	0.99983	0.250	102.71 ± 1.54
4-hydroxybenzoic acid [3]	280	1983.16 ± 13.20	362.10 ± 203.13	0.99984	0.250	109.29 ± 1.08
Caftaric acid [4]	320	2570.58 ± 12.71	1.60 ± 195.56	0.99991	0.250	115.38 ± 2.76
Catechin [5]	280	512.29 ± 5.86	102.63 ± 90.09	0.99954	0.250	103.56 ± 1.08
Vanillic acid [6]	280	2180.57 ± 12.00	336.88 ± 184.68	0.99989	0.250	106.15 ± 0.88
Caffeic acid [7]	320	7260.12 ± 29.02	745.94 ± 446.49	0.99994	0.125	106.44 ± 2.25
Chlorogenic acid [8]	320	3217.23 ± 9.61	144.97 ± 147.81	0.99997	0.125	112.55 ± 0.83
Syringic acid [9]	280	4000.64 ± 24.46	678.18 ± 376.34	0.99987	0.125	118.67 ± 3.15
Epicatechin [10]	280	904.05 ± 9.94	275.86 ± 153.00	0.99958	0.250	85.96 ± 1.66
4-coumaric acid [11]	320	7841.05 ± 36.56	1138.07 ± 562.82	0.99992	0.125	105.55 ± 0.33
Fertaric acid [12]	280	7683.78 ± 49.79	1313.76 ± 766.06	0.99985	0.125	104.44 ± 0.74
Ferulic acid [13]	320	4756.76 ± 19.29	470.95 ± 296.75	0.99994	0.125	106.77 ± 0.74
Sinapic acid [14]	320	4789.29 ± 11.47	219.64 ± 176.51	0.99998	0.250	110.93 ± 0.90
Piceid [15]	320	4972.16 ± 21.32	468.90 ± 327.95	0.99994	0.125	103.30 ± 4.50
Rutin [16]	280	956.13 ± 6.56	160.58 ± 100.99	0.99984	0.250	87.35 ± 1.92
2-hydroxy-4-methoxybenzoic acid [17]	280	1456.13 ± 14.41	219.72 ± 221.77	0.99966	0.250	107.30 ± 1.88
Resveratrol [18]	320	4657.86 ± 39.39	808.09 ± 606.06	0.99975	0.125	99.96 ± 0.85

1 Calibration range was from 0.250 to 40 mg/L, each concentration level was measured in triplicate; ^2^ Accuracy was determined as a method recovery using six spiked samples at concentration level of 5 mg/L. Precision was evaluated as the relative standard deviation of six recovery determinations in %. Blank injection of methanol was used to check the sample carry over.

### 3.3 Evaluation of the phenolic compounds profile

#### 3.3.1 Hydroxybenzoic acids

**Fig. 4:**
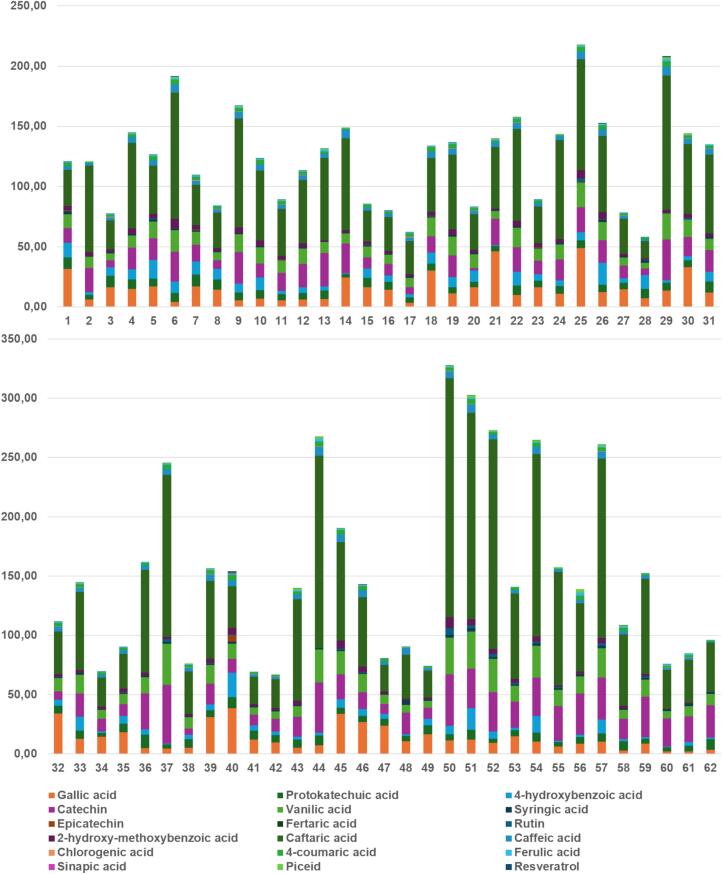
The graphical evaluation of the total phenolic profile of archival Tokaj wines. Samples 1–31 in the upper part, samples 32–62 in the bottom part. The column height responds total concentration in mg/L.

Hydroxybenzoic acids, i.e., gallic acid, protocatechuic acid, 4-hydroxybenzoic acid, syringic acid, and vanillic acid usually represent the minor group of non-flavonoid phenolic substances, which occur mainly due to the strong influence of viticultural conditions, variety, and the place of grapevine cultivation and demonstrates the significant influence of terroir, as was reported elsewhere (Kumšta, 2007; McGhie & Rowan, 2012). Despite this fact, significant variability in their concentration levels occurs. In our study, among the hydroxybenzoic acids, gallic acid, protocatechuic acid, and 4-hydroxybenzoic acid usually dominated. In agreement with that, gallic acid was quantified with the highest mean concentration of 14.65 mg/L, but unexpectedly followed by vanillic acid (13.32 mg/L), and finally by 4-hydroxybenzoic acid (6.31 mg/L) and protocatechuic acid (6.35 mg/L). Interestingly, we also compared the Tokaj wines with selected varieties of sweet white wines originating from the Czech Republic. The results presented in Table 3 confirm the dominance of Tokaj wines in terms of higher content of hydroxybenzoic acids, regardless of the variety of Czech wine, where the significantly lower values of the gallic acid (0.87–2.09 mg/L), protocatechuic (0.74–2.73 mg/L), and 4-hydroxybenzoic acid (0.36–0.86 mg/L) were found. The graphical evaluation of the hydroxybenzoic acids content including the total phenolic profile of 62 tested wines is presented in Fig. 4.

**Table 3:** Determination of eighteen phenolic compounds in the group of hydroxybenzoic acids, hydroxycinnamic acids, stilbenes, and flavanols in Tokaj archive wines in comparison with Czech wines (Kupsa et al., 2017; Lamplíř & Pavloušek, 2013).

Hydroxybenzoic acids (mg/L)									
	Putňa number						Czech wines		
Compound	3 (*n* = 8)	4 (*n* = 8)	5 (*n* = 11)	6 (*n* = 12)	Essence (n = 3)	Others (*n* = 18)	Riesling	Müller Thurgau	Chardonnay
Gallic acid	11.57 ± 7.68	11.92 ± 8.32	17.15 ± 8.33	17.22 ± 13.20	18.86 ± 13.37	13.44 ± 12.15	2.09 ± 2.11	1.21 ± 0.77	0.87 ± 0.47
Protocatechuic acid	4.86 ± 0.95	6.13 ± 1.16	7.03 ± 1.16	7.74 ± 1.43	7.73 ± 1.53	5.61 ± 2.19	2.73 ± 0.58	1.04 ± 0.03	1.71 ± 0.30
4-hydroxybenzoic acid	3.96 ± 1.58	6.99 ± 2.64	9.21 ± 3.59	11.05 ± 5.29	9.14 ± 4.56	2.10 ± 1.28	0.69 ± 0.19	0.40 ± 0.03	0.63 ± 0.11
Vanillic acid	11.84 ± 7.25	14.26 ± 7.48	12.30 ± 6.03	14.34 ± 7.05	15.83 ± 9.54	13.12 ± 7.51	0.70 ± 0.46	–	–
Syringic acid	0.87 ± 0.33	1.00 ± 0.63	1.46 ± 0.60	1.28 ± 0.64	1.40 ± 0.64	1.15 ± 0.78	0.27 ± 0.06	0.70 ± 0.59	0.85 ± 0.72
2-hydroxy-4-methoxybenzoic acid	3.01 ± 1.07	3.89 ± 2.56	3.40 ± 1.53	4.49 ± 1.96	3.39 ± 0.94	2.10 ± 1.09	–	–	–

Hydroxycinnamic acids (mg/L)									
	Putňa number						Czech wines		
Compound	3 (n = 8)	4 (n = 8)	5 (n = 11)	6 (n = 12)	Essence (n = 3)	Others (n = 18)	Riesling	Müller Thurgau	Chardonnay
Fertaric acid	<LOQ	<LOQ	<LOQ	<LOQ	<LOQ	<LOQ	3.87 ± 0.91	4.02 ± 0.82	3.20 ± 0.15
Caftaric acid	57.13 ± 51.09	71.09 ± 56.83	56.16 ± 40.17	62.98 ± 44.20	84.88 ± 61.36	69.66 ± 36.62	31.30 ± 12.95	42.52 ± 12.86	37.11 ± 7.02
Caffeic acid	3.21 ± 1.36	4.14 ± 1.39	3.60 ± 1.56	4.64 ± 1.75	5.04 ± 1.09	3.86 ± 1.96	3.10 ± 1.42	1.78 ± 0.01	1.57 ± 0.63
Chlorogenic acid	0.34 ± 0.20	0.29 ± 0.12	0.18 ± 0.06	0.31 ± 0.13	0.25 ± 0.04	0.20 ± 0.06	–	–	–
*4-*Coumaric acid	2.15 ± 0.78	2.57 ± 0.82	2.38 ± 0.89	3.18 ± 0.89	2.90 ± 0.58	2.21 ± 1.07	1.95 ± 0.54	0.34 ± 0.31	1.16 ± 0.73
Sinapic acid	<LOQ	<LOQ	<LOQ	<LOQ	<LOQ	<LOQ	–	–	–
Ferulic acid	0.54 ± 0.22	0.79 ± 0.22	0.65 ± 0.21	0.79 ± 0.19	0.74 ± 0.22	0.82 ± 0.62	0.71 ± 0.25	0.45 ± 0.18	0.50 ± 0.03

Stilbenes; Flavan-3-ols; Flavonols (mg/L)									
	Putňa number						Czech wines		
Compound	3 (n = 8)	4 (n = 8)	5 (n = 11)	6 (n = 12)	Essence (n = 3)	Others (n = 18)	Riesling	Müller Thurgau	Chardonnay
Piceid	0.50 ± 0.39	0.59 ± 0.39	0.62 ± 0.49	0.68 ± 0.58	0.62 ± 0.55	0.91 ± 0.80	0.13 ± 0.06	0.55 ± 0.23	0.11 ± 0.06
Resveratrol	0.27 ± 0.14	0.27 ± 0.08	0.31 ± 0.18	0.48 ± 0.37	0.22 ± 0.06	0.29 ± 0.21	0.18 ± 0.08	0.55 ± 0.53	0.80 ± 0.58
Epicatechin	<LOQ	<LOQ	<LOQ	0.33 ± 0.21	<LOQ	<LOQ	0.48 ± 0.22	5.86 ± 2.77	7.58 ± 1.54
Catechin	15.01 ± 8.23	19.17 ± 13.01	15.88 ± 8.19	15.62 ± 8.45	19.06 ± 12.91	25.00 ± 10.34	2.35 ± 0.67	11.87 ± 5.85	13.54 ± 3.91
Rutin	<LOQ	<LOQ	0.86 ± 0.69	0.89 ± 1.01	0.96 ± 0.91	<LOQ	–	–	–

#### 3.3.2 Hydroxycinnamic acids

Most phenolic compounds identified and quantified in Tokaj wines belong to the group of hydroxycinnamic acids derivatives, which occur in wine in significantly higher concentrations than the free form of hydroxycinnamic acids. The most abundant substances among cinnamates are esters of tartaric acid, particularly caftaric acid, which usually represents more than 50 % of the total amount of hydroxycinnamic acids in white wines (Moreno-Arribas & Polo, 2009). As expected, caftaric acid predominated among the studied phenolics, with average values of around 60 mg/L, followed by caffeic acid (3.97 mg/L) and 4-coumaric acid (2.50 mg/L). Ferulic and chlorogenic acids occurred in significantly lower concentrations of 0.74 and 0.2 mg/L, respectively, sinapic and fertaric acids could not be reliably quantified. Within the evaluation of hydroxycinnamic acids in Czech and Tokaj wines, we observed a similar trend as in the case of hydroxybenzoic acids, the higher amount of all investigated compounds in Tokaj wines with caftaric acid in the first place (17.19–42.52 mg/L), unexpectedly followed by fertaric acid (2.39–4.02 mg/L) instead of caffeic acid (1.52–3.10 mg/L). The content of 4-coumaric acid (0.34–1.95 mg/L) and ferulic acid (0.45–0.71 mg/L) was comparable in both Czech and Tokaj wines. The graphical evaluation of the content of hydroxycinnamic acids in the tested wines is presented in Fig. 4.

#### 3.3.3 Stilbenes

The primary function of stilbenes in several plant families is to act as phytoalexins. Phytoalexins are compounds synthesized predominantly in response to localized stress, such as infection by pathogens. The major phytoalexin in grapes is resveratrol (and its glycoside piceid). Phytoalexins are produced in plants to act as a defense against attacking organisms (Teka et al., 2022). Stilbenes, represented by resveratrol and its glycoside piceid, occur in white wines in significantly lower concentrations, but are not negligible due to their positive effects on human health (Guebaila et al., 2006). Resveratrol, as well as piceid, can exist in two isomeric arrangements, *cis*- and *trans*-. In this study, only the amount of the *trans-*form of both stilbenes was evaluated, which should be predominant in grapes, especially *trans-*resveratrol (Moreno-Arribas & Polo, 2009). The concentration of *trans-*resveratrol was less than 1.45 mg/L with a mean value of 0.34 mg/L, and piceid was less than 3.40 mg/L with a mean value of 0.71 mg/L. In contrast, the content of *trans-*resveratrol in Czech wines ranged from 0.12 to 0.80 mg/L with a mean value of 0.49 mg/L, and the content of piceid ranged from 0.05 to 0.55 mg/L with a mean value of 0.23 mg/L. The graphical evaluation of the content of stilbenes in the tested wines is presented in Fig. 4.

#### 3.3.4 Flavan-3-ols and flavonols

The main source of flavonoid substances in white wines is a group of flavanols, epicatechin, catechin, or flavonol rutin. In wine and the must, flavanols originate from the grape seeds and skin, so the high content of catechin in Tokaj wines in comparison with Czech wine Riesling could be due to more intensive and longer skin contact or harsher pressing during winemaking providing more effective extraction of flavanols from seeds. However, it was not confirmed for epicatechin that was found at levels lower than LOQ. In our findings, it has been observed that the higher the amount of flavanols, the higher the content of caftaric acid due to the antioxidative protection of oxidatively sensitive phenolic compounds, such as caftaric acid, which has been reported elsewhere (Pour Nikfardjam et al., 2007). Catechin was the most abundant of all flavonoids studied with concentrations ranging from 2.24 to 49.35 mg/L, and a mean of 19.24 mg/L. Epicatechin and rutin were found in significantly lower concentrations, with a mean value of 0.63 and 1.00 mg/L, respectively. A very low level of rutin fits well with the theory of its degradation in white wines as is presented elsewhere (Jeffery et al., 2008). The graphical evaluation of the content of flavanols in the tested wines is presented in Fig. 4.

### 3.4 Principal component analysis

**Fig. 5:**
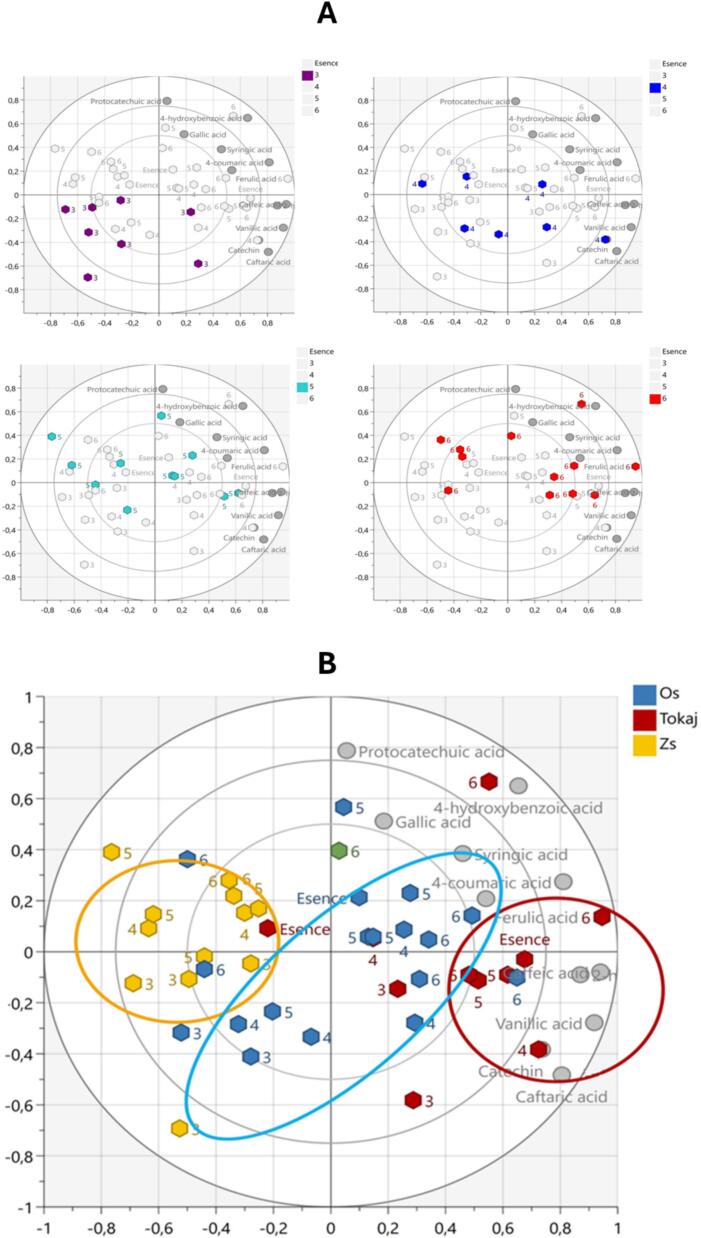
PCA - correlation between phenolic compound profile and “putňa” number (A), respectively winery (B); three clusters are based on the influence of the vinification techniques and procedures.

Nowadays, the concept of chemometric analysis, and the wide use of targeted and untargeted analytical approaches have become a trending topic, especially in the field of food and wine analysis, mostly to evaluate authenticity issues (De Angelis et al., 2024; Gomes et al., 2023; Ranaweera et al., 2021). As the first model in our study, we used the Principal Component Analysis (PCA). PCA was applied to the phenolic contents data to identify and evaluate the relationship between specific phenolic substances and the correlations between the profile of phenolic compounds and the vintage, the amount of noble rotten berries, and the different vinification techniques. The PCA was performed on the dataset containing 11 polyphenols: protocatechuic acid, catechin, 2-hydroxy-4-methoxybenzoic acid, vanillic acid, caftaric acid, ferulic acid, gallic acid, syringic acid, caffeic acid, 4-hydroxybenzoic acid, and 4-coumaric acid. The minority of phenolic compounds, which were not statistically evaluated, occurred below the limit of quantification. The set of Tokaj wines can be divided into three clusters based on the influence of the vinification techniques and procedures of each winery, and the unique climatic and pedological conditions of different vineyards. The position of each phenolic substance is determined by its effect on the respective winery (cluster). The correlation between certain phenolic compounds and the amount of noble rotten berries has been identified, but the influence of the vintage has not been recognized. With a higher putňa number, the concentration of 4-hydroxybenzoic acid, gallic acid, and syringic acid increases as shown in Fig. 5A.

The opposite position or direction of two polyphenols represents a negative correlation between these two compounds. The syringic acid, ferulic acid, 4-hydroxybenzoic acid, and 4-coumaric acid show a positive correlation, so that the concentrations of these compounds are increased or decreased, in the same wine samples. A positive correlation was also identified in the case of the group of caftaric acid, catechin, and vanillic acid, and other pairs of compounds with positive correlation are protocatechuic and gallic acid, caffeic and 2-hydroxy-4-methoxybenzoic acid. There could also be no relationship between the content of certain polyphenols, such as protocatechuic and caffeic acid, and 2-hydroxy-4-methoxybenzoic acid, because these compounds are at right angles. The greater distance of caffeic acid, 2-hydroxy-4-methoxybenzoic acid, and vanillic acid from the central point of the graph suggests a stronger influence on all three wineries. Although caftaric acid was the most abundant polyphenolic substance in all samples, vanillic acid, 2-hydroxy-4-methoxybenzoic acid, and caffeic acid also had a strong impact on the total phenolic profile of Tokaj wines as seen in Fig. 5B.

### 3.5 The soft independent modelling of class analogy

Among the various existing class modelling techniques, the first ever appeared in literature and is probably the most popular and widespread in chemometrics is Soft Independent Modelling of Class Analogy (SIMCA) (Vitale et al., 2023). SIMCA, like other class-modelling approaches, emphasizes the characterization of each class, focusing on the similarities among samples and capturing the characteristics of each class of interest. This advantage of SIMCA over other discriminant methods is a key feature that allows to have robust but simple classification models, even when data set is modified after model building (De Angelis et al., 2024). Therefore, the SIMCA model was used as the second chemometric tool in our study. The SIMCA model was built with the purpose of authenticating botrytized Tokaj samples with respect to non-botrytized ones using phenolic content data, since the former group holds higher economic value. First, the data were partitioned into training (25 botrytized samples) and test sets (14 botrytized and 21 non-botrytized samples) via the Kennard-Stone approach (Gomes et al., 2023). Only botrytized samples were included in the training stage, and therefore, the models were developed in rigorous mode. The significance level for accepting samples as belonging to the modeled target class was set at 5 %, while a 1 % threshold was used for outlier detection. A full description of this approach can be found elsewhere (Vitale et al., 2023).

**Fig. 6:**
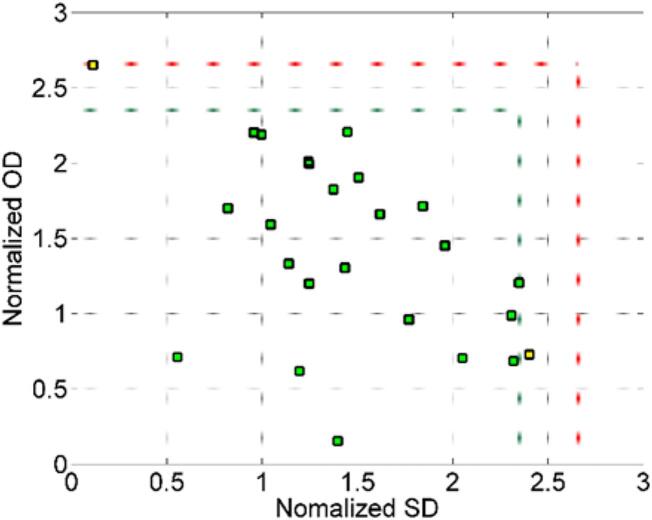
Training set acceptance area. The green and red dotted lines define the limits of the regular samples accepted in the target class with α = 0.05 and samples considered outliers with γ = 0.01. Green, yellow, and red squares are the regular, extreme, and outlier samples respectively. (For interpretation of the references to colour in this figure legend, the reader is referred to the web version of this article.)

The results demonstrated in the Supplementary material section Table 3S display the cumulative variance as well as the training sensitivity for different numbers of principal components (PCs) in the SIMCA model. As can be observed, beyond the 4th principal component no significant gains in variance are achieved, while the sensitivity approaches the 95 % threshold adopted as the significance level during training. Therefore, this number of PCs was applied to the test set to validate this selection. The acceptance area obtained with 4 PCs is presented in Fig. 6. Only 2 wine samples in the training set were rejected within the class space defined by the orthogonal distance (OD) and score distance (SD), yet none of them were considered outliers.

**Fig. 7:**
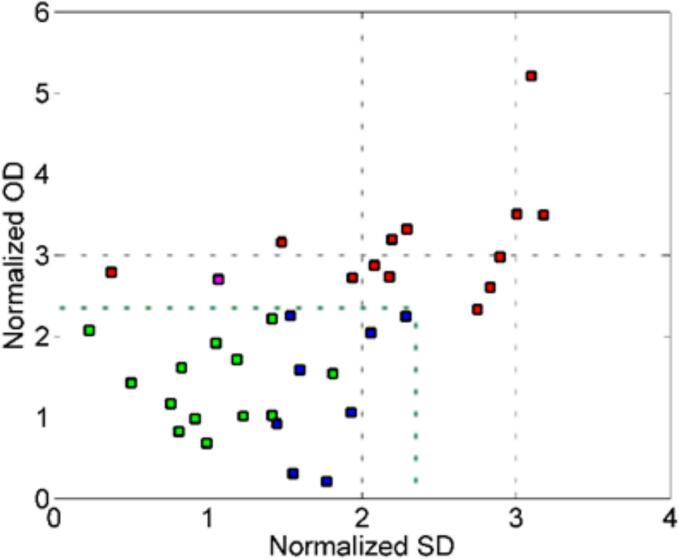
Test set projection in the acceptance area (green line corresponds to α = 0.05) defined in the training stage. Green, red, and blue squares are true positives, true negatives, and false positives respectively. (For interpretation of the references to colour in this figure legend, the reader is referred to the web version of this article.)

When applied to the test set, the model demonstrated performance with 93 % sensitivity, 62 % specificity, resulting in an overall efficiency of 75 %. As can be seen in Fig. 7, the projection of the test samples within the acceptance region. Analysis of phenolic compounds' influence on the SIMCA model identified these key contributors from phenolic profile to botrytized/non-botrytized Tokaj wine sample discrimination: the content of gallic acid, 4-hydroxybenzoic acid, catechin, and caftaric acid in Tokaj wines.

### 3.6 Determination of total antioxidant activity

The total amount of antioxidants in archive wines, Tokaj selections 3–6 putňa, Tokaj Essences, and other wines was evaluated using the flow analysis method coupled with a CoulArray electrochemical detector. This method allows the antioxidant characterization of Tokaj wines in a short time. The total time of analysis was 70 s due to the absence of a separation step. In contrast, the absence of a chromatography column makes it impossible to determine the antioxidant activity of each individual phenolic substance. The total content of all phenolic compounds (mg/L) and the antioxidant capacity of the wines expressed in μC are reported in the Supplementary Material Table 2S. A significant correlation (r^2^ = 0.6457) between the sum of all phenolic substances (in mg/L) measured by UHPLC-DAD and the total antioxidant activity of Tokaj selections (3–6 putňa) via FIA-CoulArray (in μC) is presented in Fig. 1S in Supplementary Material. We observed that the increasing putňa number of selections increased the total antioxidant activity, probably due to the enhanced content of stilbenes and gallic acid. Gallic acid is the most common equivalent chemical standard for antioxidant capacity determination because of 3 hydroxy groups in the molecule. Nevertheless, the total antioxidant activity can be affected by caftaric acid, which predominates in the phenolic profile and contain two hydroxy groups. Further major phenolic acids, 4-hydroxybenzoic acid, vanillic acid, and protocatechuic acid with one or two hydroxy-groups contributed to enhanced antioxidant capacity. Phytoalexins stilbenes that were found in wine as the metabolic response of the plant to noble rot infection and defense mechanism against pathogenic attack and UV radiation well correlated with increasing antioxidant capacity and number of putňa 3–6. However, the relationship between the Tokaj essences, other types of Tokaj wines, and total antioxidant activity was not identified probably due to the high sugar content and statistically insignificant sample set (3 samples of Tokaj essence), or due to the lack of satisfactory characterization of the samples (unidentified Tokaj selections).

## 4 Conclusion

The profile of phenolic compounds can provide relevant information for assessing wine authenticity and can be successfully used to determine the geographical origin. Therefore, we developed a new UHPLC-DAD method for the rapid analysis and simultaneous quantification of eighteen phenolic compounds in Tokaj wines originating from the Slovak part of the Tokaj region. A Kinetex Polar C18 core-shell analytical column enabled the separation of the vast majority of the investigated phenolic compounds within 13 min. The minority of them were eluted in up to 18 min. The results of the phenolic profile evaluation indicated that caftaric acid with a mean value of 65.28 mg/L was the most abundant phenolic compound in the tested wines, followed by catechin with a mean value of 19.24 mg/L, gallic acid with a mean value of 14.65 mg/L, and vanillic acid with a mean value of 13.32 mg/L. Chemometrics plays an indispensable role in the interpretation and modelling of analytical data and is frequently used for sample classification and wine authentication (Ranaweera et al., 2021). In our study, we used two individual statistical approaches for the robust classification of Tokaj wines – PCA and SIMCA model. PCA statistical evaluation of the phenolic profile provided information on the positive correlation between syringic acid, ferulic acid, 4-hydroxybenzoic acid, and 4-coumaric acid. The relationship between the phenolic profile and wineries, their specific climatic conditions, and vinification techniques was identified. In contrast, the relationships among the profiles of phenolic compounds and the vintage were not recognized. Certain correlations were identified between the selected phenolic compounds, and the amount of nobble rotten berries to putňa number ranged from 3 to 6. In particular, the concentration of gallic, 4-hydroxybenzoic acid, and syringic acid increased significantly with the number of nobble-rotten berries. SIMCA model of phenolic compounds' influence identified the main contributors to distinguish the botrytized and non-botrytized Tokaj wines. Namely, the concentration of gallic acid, 4-hydroxybenzoic acid, catechin, and caftaric acid in archival Tokaj wines. Together, PCA and SIMCA model showed the significant influence of 4-hydroxybenzoic and gallic acid to distinguish botrytized and non-botrytized wines and putňa numbers. In addition, the results of our study proved that a higher putňa number of Tokaj selections provided higher total antioxidant activity. The evaluation of a wide range of Tokaj archive wines and their characterization based on the phenolic profile is one of the first studies of its kind. As was presented elsewhere (Furdíková et al., 2020; Furdíková et al., 2021; Khvalbota et al., 2021a; Machyňáková et al., 2019; Sádecká & Jakubíková, 2020; Vyviurska & Špánik, 2020), these wines were so far characterized based on volatile organic compounds only. Thus, this study contributes to completing the chemical characterization of archival Tokaj wines.

## CRediT authorship contribution statement

**Pavlína Moravcová:** Writing – original draft, Visualization, Validation, Project administration, Methodology, Formal analysis. **Ivan Špánik:** Visualization, Resources. **Jan Škop:** Formal analysis. **Aleš Horna:** Data curation. **František Švec:** Writing – review & editing, Formal analysis. **Adriano de Araújo Gomes:** Software, Formal analysis, Data curation. **Dalibor Šatínský:** Writing – review & editing, Supervision, Resources, Investigation, Funding acquisition, Conceptualization.

## Declaration of competing interest

The authors declare that they have no known competing financial interests or personal relationships that could have appeared to influence the work reported in this paper.

## Data Availability

Data will be made available on request.

## Appendix A Supplementary data

**Table 1S** List of evaluated archive Tokaj wines. **Table 2S** Sum of all phenolic substances (in mg/L) measured via UHPLC-DAD and total antioxidant capacity determined by flow injection analysis (FIA) coupled with CoulArray electrochemical detector (in μC), data are mean of three replications ± standard deviation. **Figure 1S** Correlation between total phenolics (mg/L) and total antioxidant activity (μC). **Table 3S** The cumulative variance (CV) and training sensitivity (SENtrain) versus the number of principal components (PCs). Image 1
