# Supplementary material for: The profiling of phenolic compounds in archive Tokaj wines using liquid chromatography and antioxidant activity analysis
Source: Food Chem X. 2025 Aug 7;30:102897. doi: 10.1016/j.fochx.2025.102897 (PMC12359266; doi:10.1016/j.fochx.2025.102897)
Supplement: Supplementary file 1 — Table 1S List of evaluated archive Tokaj wines. Table 2S Sum of all phenolic substances (in mg/L) measured via UHPLC-DAD and total antioxidant capacity determined by flow injection analysis (FIA) coupled with CoulArray electrochemical detector (in μC), data are mean of three replications ± standard deviation. Figure 1S Correlation between total phenolics (mg/L) and total antioxidant activity (μC). Table 3S The cumulative variance (CV) and training sensitivity (SENtrain) versus the number of principal components (PCs). [file mmc1.docx]

# **Supplementary material**

# **The profiling of phenolic compounds in archive Tokaj wines using liquid chromatography and antioxidant activity analysis**

Pavlína Moravcová ^1^, Ivan Špánik ^2^, Jan Škop^1^, Aleš Horna ^1,3^, František Švec ^1^, Adriano de Araújo Gomes ^4^, Dalibor Šatínský ^1*^

^1^ Department of Analytical Chemistry, Faculty of Pharmacy in Hradec Králové, Charles University, Ak. Heyrovského 1203, Hradec Králové 500 05, Czech Republic

^2^ The Institute of Analytical Chemistry, Faculty of Chemical and Food Technology, the Slovak University of Technology in Bratislava, Radlinského 9, Bratislava 812 37, Slovakia

^3^ Institute of Nutrition and Diagnostics Pardubice, Sakařova 1400, 530 03 Pardubice, Czech Republic

^4^ Universidade Federal do Rio Grande do Sul, Instituto de Química, 90650-001, Porto Alegre, RS, Brazil

*Corresponding author:

Dalibor Šatínský

E-mail address: [satinsky@faf.cuni.cz](mailto:satinsky@faf.cuni.cz)

Table 1S List of evaluated archive Tokaj wines

|  | **Tokaj wine** | **Puttony** | **Vintage** | **Winery** |
| --- | --- | --- | --- | --- |
| 1 | Archiv wine | 5 | 1983 | Zlatý Strapec |
| 2 | Archiv wine | 3 | 2003 | Ostrožovič |
| 3 | Tokaj Archiv wine | 6 | 1972 | Zlatý Strapec |
| 4 | Archiv wine | 5 | 2004 | Ostrožovič |
| 5 | Archiv wine | 5 | 1989 | Ostrožovič |
| 6 | Archiv wine | 6 | 2002 | Ostrožovič |
| 7 | Tokaj Archiv wine | 6 | 1983 |  |
| 8 | Archiv wine | 6 | 1993 | Zlatý Strapec |
| 9 | Archiv wine | 4 | 2004 | Ostrožovič |
| 10 | Archiv wine | 4 | 2002 | Ostrožovič |
| 11 | Archiv wine | 3 | 1995 | Ostrožovič |
| 12 | Archiv wine | 4 | 1999 | Ostrožovič |
| 13 | Tokaj | 4 | 2016 | Ostrožovič |
| 14 | Šupkáš wine with prolonged maceration (90 days) | - | 2017 |  |
| 15 | Archiv wine | 6 | 1997 | Zlatý Strapec |
| 16 | Archiv wine | 5 | 1993 | Zlatý Strapec |
| 17 | Archiv wine | 3 | 1999 | Ostrožovič |
| 18 | Archiv wine | 4 | 1995 | Tokaj&Co |
| 19 | Archiv wine | 6 | 1993 | Ostrožovič |
| 20 | Tokaj selection | 4 | 1993 | Zlatý Strapec |
| 21 | Furmint-Šupkáč 30 days maceration | - | 2017 |  |
| 22 | Tokaj selection | 6 | 2003 | Ostrožovič |
| 23 | Archiv wine | 3 | 1988 | Zlatý Strapec |
| 24 | Archiv wine | 5 | 2003 | Ostrožovič |
| 25 | Archiv wine | 6 | 1993 | Tokaj&Co |
| 26 | Archiv wine | 6 | 1989 | Ostrožovič |
| 27 | Tokaj selection | 3 | 1995 | Zlatý Strapec |
| 28 | Archiv wine | 5 | 1959 | Zlatý Strapec |
| 29 | Muskat yellow, Cibeba selection | - | 2012 | Ostrožovič |
| 30 | Archiv wine Samorodné sladké | - | 1997 | Tokaj&Co |
| 31 | Archiv wine Essence | >6 | 2000 | Ostrožovič |
| 32 | Archiv wine Essence | >6 | 1999 | Tokaj&Co |
| 33 | Archiv wine | 5 | 1993 | Ostrožovič |
| 34 | Archiv wine Samorodné suché | - | 1997 | Zlatý Strapec |
| 35 | Archiv wine | 5 | 1972 | Zlatý Strapec |
| 36 | Muskat yellow, Straw wine | - | 2010 | Ostrožovič |
| 37 | Muskat yellow | - | 2015 | Ostrožovič |
| 38 | Archiv wine | 6 | 1999 | Ostrožovič |
| 39 | Selection | 2 | 1990 | Tokaj&Co |
| 40 | Archiv wine | 6 | 1989 | Tokaj&Co |
| 41 | Archiv wine | 4 | 2000 | Zlatý Strapec |
| 42 | Selection | 3 | 2000 | Zlatý Strapec |
| 43 | Furmint, Cibeba selection | - | 2013 | Ostrožovič |
| 44 | Furmint, Cibeba selection | - | 2012 | Ostrožovič |
| 45 | Archiv wine | 5 | 1990 | Tokaj&Co |
| 46 | Archiv wine | 3 | 1990 | Tokaj&Co |
| 47 | Archiv wine | 2 | 1989 | Tokaj&Co |
| 48 | Archiv wine Samorodné suché | - | 2016 | Ostrožovič |
| 49 | Tokaj selection | 5 | 2000 | Zlatý Strapec |
| 50 | Tokaj selection | 4 | 2009 | Tokaj&Co |
| 51 | Tokaj selection | 6 | 2006 | Tokaj&Co |
| 52 | Tokaj selection | 3 | 2009 | Tokaj&Co |
| 53 | Tokaj Forditáš | - | 2011 | Tokaj&Co |
| 54 | Tokaj Essence | >6 | 2009 | Tokaj&Co |
| 55 | Archiv wine Samorodné sladké | - | 2006 | Tokaj&Co |
| 56 | Lipovina | - | 2015 | Ostrožovič |
| 57 | Tokaj selection | 5 | 2003 | Tokaj&Co |
| 58 | Furmint | - | 2014 | Ostrožovič |
| 59 | Archiv wine Samorodné suché | - | 2009 | Tokaj&Co |
| 60 | Archiv wine Samorodné suché | - | 2015 | Tokaj&Co |
| 61 | Muskat yellow polosladké | - | 2015 | Zlatý Strapec |
| 62 | Tokaj samorodné suché | - | 2015 | Tokaj&Co |

Table 2S Sum of all phenolic substances (in mg/L) measured via UHPLC-DAD and total antioxidant capacity determined by flow injection analysis (FIA) coupled with CoulArray electrochemical detector (in μC), data are mean of three replications ± standard deviation.

|  | **Putňa index** | | | |  |  |
| --- | --- | --- | --- | --- | --- | --- |
| **Phenolic compounds (mg/L)** | **3 (n= 8)** | **4 (n=8)** | **5 (n=11)** | **6 (n=12)** | **Essence (n=3)** | **Others (n=18)** |
| **Total content** range (mg/L) | 62.33-273.16 | 69.35-327.65 | 58.28-261.27 | 76.56-302.57 | 111.69-264.69 | 69.72-267.64 |
| **Total content** mean value (mg/L) ± SD | 115.45±69.24 | 143.86±80.27 | 130.59±58.26 | 145.67±67.70 | 170.53±82.39 | 140.54±53.48 |
| **Antioxidant capacity** (µC) | 0.46±0.08 | 0.51±0.10 | 0.58±0.09 | 0.65±0.30 | 0.55±0.06 | 0.39±0.08 |

Figure 1S Correlation between total phenolics (mg/L) and total antioxidant activity (μC).

Table 3S The cumulative variance (CV) and training sensitivity (SENtrain) versus the number of principal components (PCs).

| **PCs** | **CV** | **SENtrain** |
| --- | --- | --- |
| 1 | 93.287 | 0.840 |
| 2 | 98.415 | 0.880 |
| 3 | 99.246 | 0.875 |
| **4** | **99.777** | **0.920** |
| 5 | 99.926 | 0.864 |
| 6 | 99.967 | 0.957 |
| 7 | 99.990 | 0.913 |
| 8 | 99.996 | 0.952 |
| 9 | 99.999 | 0.957 |
